# Heavy metals from non-exhaust vehicle emissions in urban and motorway road dusts

**DOI:** 10.1007/s10661-016-5377-1

**Published:** 2016-05-26

**Authors:** Ewa Adamiec, Elżbieta Jarosz-Krzemińska, Robert Wieszała

**Affiliations:** Faculty of Geology, Geophysics and Environmental Protection, AGH University of Science and Technology, Al. A. Mickiewicza 30, 30-059 Kraków, Poland; Faculty of Transport, SUT Silesian University of Technology, ul. Akademicka 2A, 44-100 Gliwice, Poland

**Keywords:** Heavy metal, Road dust, Brake pad, Tire, Geo-accumulation index

## Abstract

The main sources of non-exhaust vehicular emissions that contribute to road dust are tire, brake and clutch wear, road surface wear, and other vehicle and road component degradation. This study is an attempt to identify and investigate heavy metals in urban and motorway road dusts as well as in dust from brake linings and tires. Road dust was collected from sections of the A-4 motorway in Poland, which is part of European route E40, and from urban roads in Katowice, Poland. Dust from a relatively unpolluted mountain road was collected and examined as a control sample. Selected metals Cd, Cr, Cu, Ni, Pb, Zn, Fe, Se, Sr, Ba, Ti, and Pd were analyzed using inductively coupled plasma-mass spectrometry, inductively coupled plasma (ICP)-optical emission spectroscopy, and atomic absorption spectroscopy on a range of size-fractionated road dust and brake lining dust (<20, 20–56, 56–90, 90–250, and >250 μm). The compositions of brake lining and tire dust were also investigated using scanning electron microscopy-energy-dispersive spectroscopy. To estimate the degree of potential environmental risk of non-exhaust emissions, comparison with the geochemical background and the calculations of geo-accumulation indices were performed. The finest fractions of urban and motorway dusts were significantly contaminated with all of the investigated metals, especially with Ti, Cu, and Cr, which are well-recognized key tracers of non-exhaust brake wear. Urban dust was, however, more contaminated than motorway dust. It was therefore concluded that brake lining and tire wear strongly contributed to the contamination of road dust.

## Introduction

Heavy metals from vehicular emissions can be significant threats to humans and the environment because they have adverse effects on ecosystems inducing contamination of air, water, and soil. Dust generated during the operation of vehicles is derived from a number of sources, such as wearing of breaking systems, tires and clutch plates, erosion of the active layer of the catalytic converter, or resuspension of road dust. Mechanical and chemical weathering of pavement and shoulders, as well as windblown soil and dust from the surroundings, is considered a natural source of dust.

Recently, Gunawardana et al. (2012) have shown that road dust primarily consisted of soil-derived minerals (60 %), where 40–50 % of the soil-derived minerals were quartz. The remaining amount was clay-forming minerals such as albite, microcline, chlorite, and muscovite. Organic matter from plants constituted approximately 2 % of road dust, and the remaining amount originated from brake and tire ware, combustion emissions, and fly ash from asphalt. A sandpaper-like effect on road asphalt and bitumen can cause additional contamination because of the abrasion of white, yellow, and red paints used on roads and the grey paint and anticorrosive coatings used on guardrails.

Due to significant variability, identifying the chemical composition of road dust from vehicles is complex. The growing automotive industry and more restrictive environmental requirements have supported rapid progress in materials science development. Each year, numerous new materials are introduced into the automotive industry marketplace. More than 100 formulations of patented friction materials used in braking systems have existed since 1978 (Newman 1978). Presently, the number of materials used in braking systems is difficult to estimate. A wide variety of components are commonly used in vehicle brake lining, from steel or glass fibers and plastics that serve as reinforcements to brass chips that are used for their heat-conducting properties (Chan and Stachowiak 2004). This wide variety of materials contributes to the complexity of non-exhaust vehicle emissions.

Well-recognized sources of road dust contamination are tire wear and road surface abrasion. Detailed analyses of tire wear dust have previously been conducted by Fukuzaki et al. (1986), Fauser et al. (1999), Adachia and Tainoshob (2004), Schauer et al. (2006), and Hjortenkrans et al. (2007). These studies confirmed that significant amounts of Zn, Cd, Co, Cr, Cu, Hg, Mo, Ni, and Pb were associated with dust from tire wear. Zn was the most abundant heavy metal from tire wear. Its high concentrations resulted from the addition of ZnO and ZnS to the tire during vulcanization. According to Ozaki et al. (2004), tires contain approximately 1.3–1.7 % Zn, but Smolders and Degryse (2002) have reported that tires contained between 0.4 and 4.3 % Zn. Other studies have attempted to estimate emissions from tire wear and reported the emissions to range between 16 and 90 mg/tire/km (Baekken 1993; Lee et al. 1997; Legret and Pagotto 1999). The average mass of a new car tire is approximately 8 kg, and during its lifetime, it loses up to 1.5 kg. This means that within 3 years, 10–20 % of rubber enters the environment due to abrasion. The greatest wear occurs during acceleration, braking, and cornering.

Asphalt and sandpaper-like effects are significant sources of Ni and As in road dust (Ozaki et al. 2004). Gadd and Kennedy (2000) have reported that the concentrations of Ni and Zn in road bitumen were higher than in raw bitumen. This suggests that heavy metal concentrations in road dust are significantly affected by vehicle operation and road abrasion. It should be noted that more tire abrasion occurs when a vehicle drives on a concrete motorway compared with an asphalt surface (Duong and Lee 2011). Driving on concrete surfaces also requires higher energy use, which results in higher fuel consumption. Higher hydrocarbon concentrations and lower heavy metal concentrations were reported from driving on asphalt. Heavy metal concentrations in road dust strongly depend on vehicle speed. As such, the highest concentrations have been recorded on motorways. Higher speeds also result in greater tire wear and increased fuel combustion. Duong and Lee (2011) compared dust from roads, where the average speed ranged from 80 to 90 km/h with roads where the average speed ranged from 70 to 80 km/h. They have found that higher concentrations of heavy metals occurred in dust from roads that had higher average driving speeds. According to Duong and Lee (2011), the concentrations of heavy metals in road dust vary significantly depending on traffic and road features such as roundabouts, motorway roads, and traffic lights. The concentrations of metals in road dust from motorways are approximately twice those found near roundabouts and downtown areas (Duong and Lee 2011). The influence of different pavement surfaces on environmental heavy metal pollution has recently been investigated by Murphy et al. (2015).

Another source of road dust is wear from braking systems. During rapid braking, brakes are exposed to extensive heat from friction, which is transmitted to the brake discs and results in the emission of particles. The most intense brake wear occurs at intersections, corners, traffic lights, and through forced braking. According to Österle et al. 2001, standard brake linings consist of 48 % barite, 14 % vermiculite, 19 % phenolic resin, 4.6 % antimonite, 5 % rubber, 6.4 % aramide, and 0.3 % sulfur. The composition, function, and friction testing of brake materials and their additives have been reported in detail (Blau 2001). Adachia and Tainoshob (2004) and Hjortenkrans et al. (2007) have reported that brake dust mainly contained not only Fe, but also significant amounts of Cu, Sb, Ba, Al, Si, S, Ti, Zn, Ni, Cr, and Pb and a small amount of Cd.

Exhaust emissions and their role in air quality are widely recognized (Kittelson 1998; Burtscher 2005; Maricq 2007; Ali and Athar 2008; Beelen et al. 2008; Biswas et al. 2009; Walsh 2011). The automotive industry has been forced to implement pollution reduction mechanisms since the European Commission established set of European guidelines about light-duty vehicles and vehicles >3.5 t. These guidelines have led to improvements in exhaust emission control technologies, but non-exhaust particle emissions remain high. There is wide societal interest in establishing a new set of regulations for non-exhaust emissions because they are non-existent. To provide critical information about non-exhaust emissions, it is necessary to broaden existing knowledge through comprehensive research programs that focus on sources of traffic pollution such as tire wear, brake wear, road surface wear, corrosion, and resuspension of road dust. Previous studies on non-exhaust emissions are insufficient because most of them have only focused on determining metal concentrations in bulk road dust samples (Kabadayi and Cesur 2010; Guney et al. 2010; Chen et al. 2012). Only few studies have focused on the particle size distribution of road dust (e.g., Ewen et al. 2009; Grigoratos and Martini 2015; Vu et al. 2015), and Dongarrà et al. (2009) attempted to analyze brake dust in air particulate matter pollution. Additionally, there is still insufficient information about the differentiation between dusts from wearing of car parts and of geogenic origin.

The main objective of this study was to quantify heavy metal concentrations within different road dust size fractions. Research involved studying the chemical and mineralogical characteristics of urban and motorway road dusts and characterized brake lining and tire dust. Analyses of dust from wearable parts of vehicle were performed to determine the influence of these non-exhaust emission sources on the concentrations of heavy metals in road dust. Comparisons of urban and motorway dusts with mountain road dust were performed, and the geo-accumulation index (*I*_geo_) was calculated to evaluate the level of road dust contamination.

## Materials and methods

### Study area

**Fig. 1:**
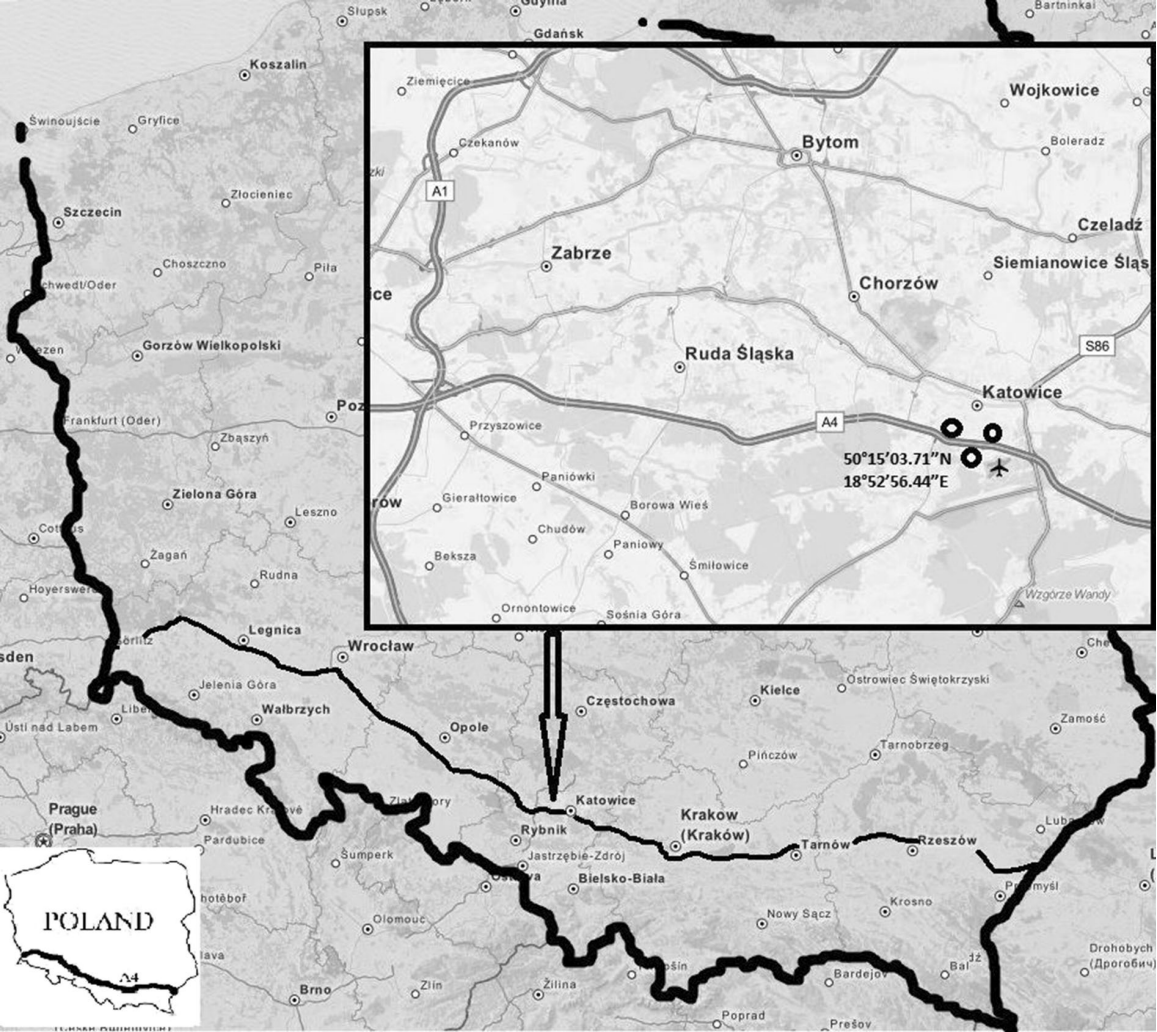
Sampling locations

Road dust was collected from urban roads in Katowice, Poland, on sections of the A-4 Katowice-Chorzów Batory motorway and from a relatively unpolluted mountain area in the Krowiarki Pass (Żywiecki Beskid, Poland). The sampling areas are presented in Fig. 1. The A-4 motorway is part of European route E40, which connects to France via Belgium, Germany, Poland, the Ukraine, and Russia to the border of China.

The sampling locations were selected to minimize the impact of other non-traffic related contamination sources. They were located as far as possible from industrial plants and, in the case of the motorway, far from residential areas. Samples were collected during spring and summer to avoid the impact of other non-traffic-related pollutants, such as those emitted from household furnaces. Section of the examined A4 motorway was straight, with no more than 3 % slope, approx. 30-m width with asphalt pavement surface and curbs on each side of the road of about 15-m width. Prior sampling, there was no rainfall for 4 days and temperature during sample collection was between 19 and 24 ° C. An average vehicle speed measured on the A-4 Katowice-Chorzów Batory motorway was 103 ± 4 km/h (cars), 92 ± 3 km/h (vans), 88 ± 3 km/h (trucks), and 89 ± 4 km/h (buses). An average vehicle speed in the Panewnicka-Katowice city center was approximately 35 ± 6 km/h for each vehicle type, and average speed in the Krowiarki Pass was approximately 56 ± 9 km/h for each vehicle type.

Samples of road dust, including field duplicates, were collected in situ using a vacuum cleaner specifically modified for collecting road dust.

### Methods

Brake lining dust was collected in automotive repair station directly from disc and drum brake pads.

Brake lining, tire, and road dust samples were sieved into five size fractions (<20, 20–56, 56–90, 90–250, and >250 μm). Twenty samples of each dust type were sieved, and Student’s *t* tests were performed for comparisons.

The compositions of brake lining and tire dust were conducted in Materials Science Laboratory at the Faculty of Materials Engineering and Metallurgy, SUT Silesian University of Technology, using scanning electron microscopy (SEM) (Hitachi S4200). The experimental conditions were as follows: a 15-keV primary electron beam, a cold cathode with field emission, and an absorption current of 1 × 10^−10^ A. The signal of secondary electrons was used, and magnifications from ×50 to ×8000 were applied for microphotography of the dust samples. An X-ray spectrometer (Voyager, NORAN (detector Si–Li, thin polymer window) for energy-dispersive spectroscopy (EDS) was used to determine the chemical composition of the dust.

Metals were extracted from brake lining dust by microwave oven digestion with aqua regia, and road dust was digested according to the US EPA method 3050B (EPA 1996). Analysis was performed according to the standard certified analytical quality control procedure (PN-EN ISO 17294-1:2007) at the Faculty of Geology, Geophysics and Environmental Protection, AGH University of Science and Technology. Cd, Cr, Cu, Ni, Pb, Se, Sr, Zn, Fe, Ba, Ti, and Pd were then quantified using inductively coupled plasma-mass spectrometry (ICP-MS) (ELAN 6100; Perkin Elmer), according to the US EPA method 6020B (EPA 1998). The concentrations of metals in road dust were then compared with the local geochemical background for Upper Silesia based on Lis and Pasieczna (1995), and the *I*_geo_ index was calculated according to Müller (1969). *I*_geo_ provides information about the level of metal accumulation and was calculated from Eq. 1 (Salomons and Förstner 1984):

1 $$ {I}_{geo}=lo{g}_2\cdot \left(\frac{C_n}{1.5\cdot Bn}\right), $$

where *C*_*n*_ is the concentration of element *n* and *B*_*n*_ is the geochemical background.

### Data quality

To obtain unambiguous and unbiased ICP-MS results, Cd, Cr, Cu, Fe, Ni, Se, Sr, Pd, Ba, and Ti were also measured using inductively coupled plasma-optical emission spectroscopy (ICP-OES) (OPTIMA 7300DV; Perkin Elmer) and Fe, Zn, Pb, Cu, and Cd were measured using atomic absorption spectroscopy (AAS) (F-AAS Thermo Scientific IC 3500), according to US EPA method 7000 (EPA 1998) in the Laboratory of Trace Analyses at AGH University of Science and Technology. To estimate the accuracies and biases of the analytical methods, reagent blanks and certified international reference materials BCR 723 (EU JRC Institute for Reference Materials and Measurements) and METRANAL™1 (Analytika, Czech Republic) were used to ensure that the analytical results met the required criteria. Analyses of the reference materials verified and confirmed the quality of the results. Analytical bias was statistically insignificant (*p* = 0.05), and the precisions of AAS and ICP-MS systems were satisfactory, which was verified by six different solution injections. Rh was used as an internal standard. Using ICP-MS, element correction equations were used for each element to minimize the impact of interferences.

## Results and discussion

### Characteristics of dust from brake linings and tires

**Fig. 2:**
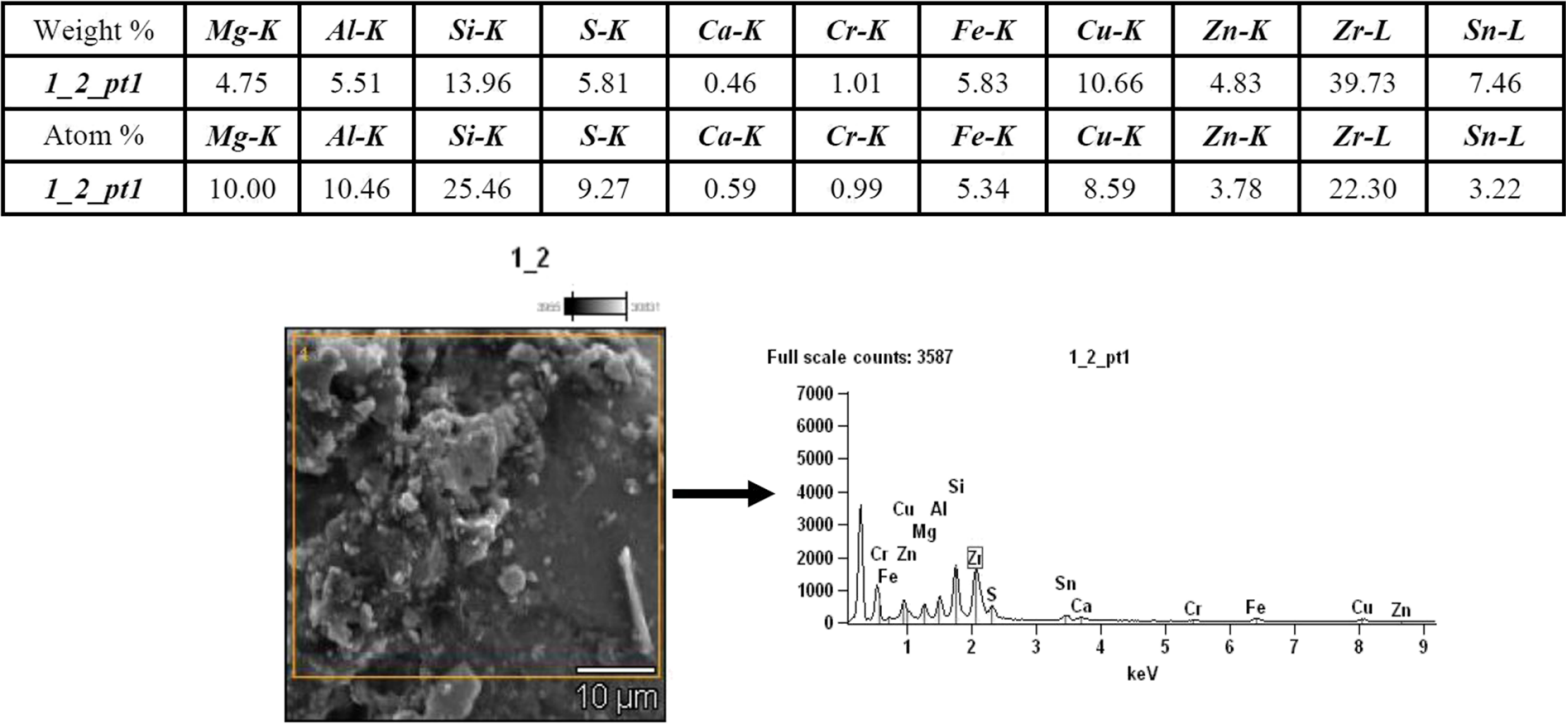
Results from SEM-EDS analyses of brake lining dust smaller than 20 μm in diameter (accelerating voltage = 15.0 kV; magnification = ×2000)

**Fig. 3:**
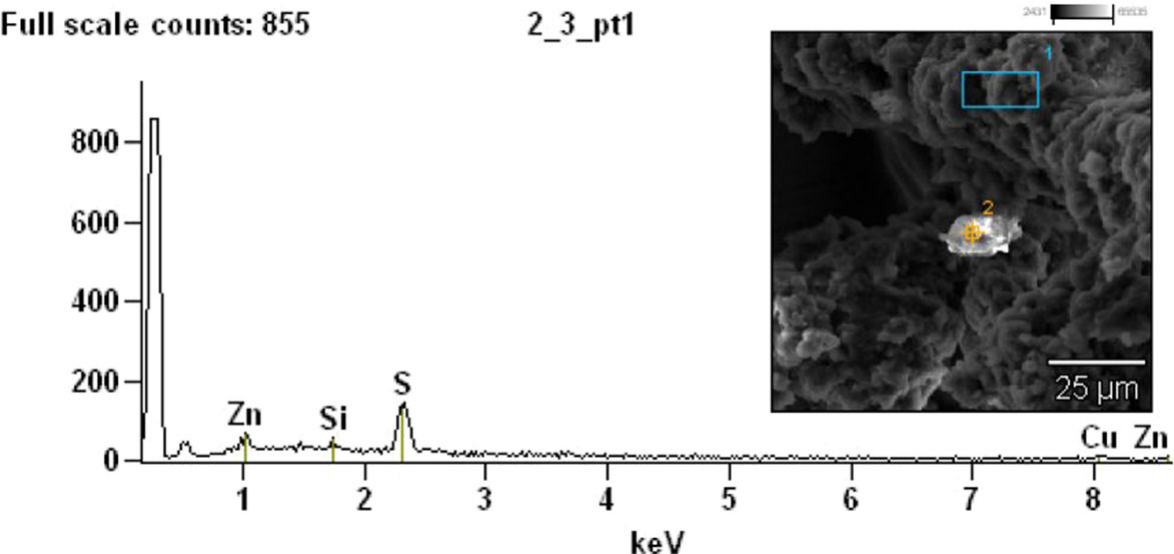
Microphotograph of tire dust smaller than 20 μm at magnification of ×1000

Results from SEM-EDS analyses conducted on the dust fractions less than 20 μm from brake linings and tires are presented in Figs. 2 and 3, respectively. Results revealed the presence of Zn, S, Si, and Cu in tire dust and Cr, Fe, Zn, Cu, Mg, Al, Si, Zr, S, and Ca in brake lining dust.

EDS analysis of brake lining dust confirmed the presence of zirconium silicate, ZrSiO_4_, which is a friction modifier (a filler for increasing friction resistance). Results from tire dust analyses, depicted on Fig. 3, confirmed the presence of ZnO and ZnS, which are added to activate vulcanization in the tire tread.

Total concentrations of Cr, Zn, Pb, Ni, Cu, Ti, Sr, Ba, Se, Cd, Fe, and Pd in bulk and size-segregated brake lining dust samples are presented in Table 1. Concentrations of the metals varied significantly within the fractions. Analysis of the bulk sample revealed that the brake dust was heavily contaminated, especially with Fe (184,780 mg/kg), Ba (20,900 mg/kg), Ti (25,400 mg/kg), Cu (14,002 mg/kg), Zn (8310 mg/kg), Pb (2897 mg/kg), and Cr (789 mg/kg). The highest concentrations of investigated metals were found in the smallest fractions (<20 and 20–56 μm) of brake dust rather than in the coarser fractions (56–90 and 90–250 μm).

**Table 1:** Concentrations of heavy metals in bulk and size-segregated brake lining dust samples

Elements	Weight %	Cr	Zn	Fe	Pb	Ni	Cu	Ti	Sr	Ba	Se	Cd	Pd
Mean concentration in brake pads dust (mg/kg)													
Bulk sample	100	789	8310	184,780	2897	369	14,002	25,400	156	20,900	7.88	2.34	0.082
Mean concentration in fraction size of brake pads dust (mg/kg)													
<20 μm	87.38	920	8763	208,099	2656	433	17,404	–	189	–	11.3	2.39	0.108
20–56 μm	6.83	899	6728	199,009	2902	456	16,309	–	166	–	9.8	2.21	0.083
56–90 μm	3.92	310	1490	169,950	928	56.3	301	–	20.5	–	1.02	0.98	0.022
90–250 μm	1.87	87.9	1039	160,358	789	20	158	–	18.1	–	0.46	0.65	0.020

### Road dust characteristics

**Fig. 4:**
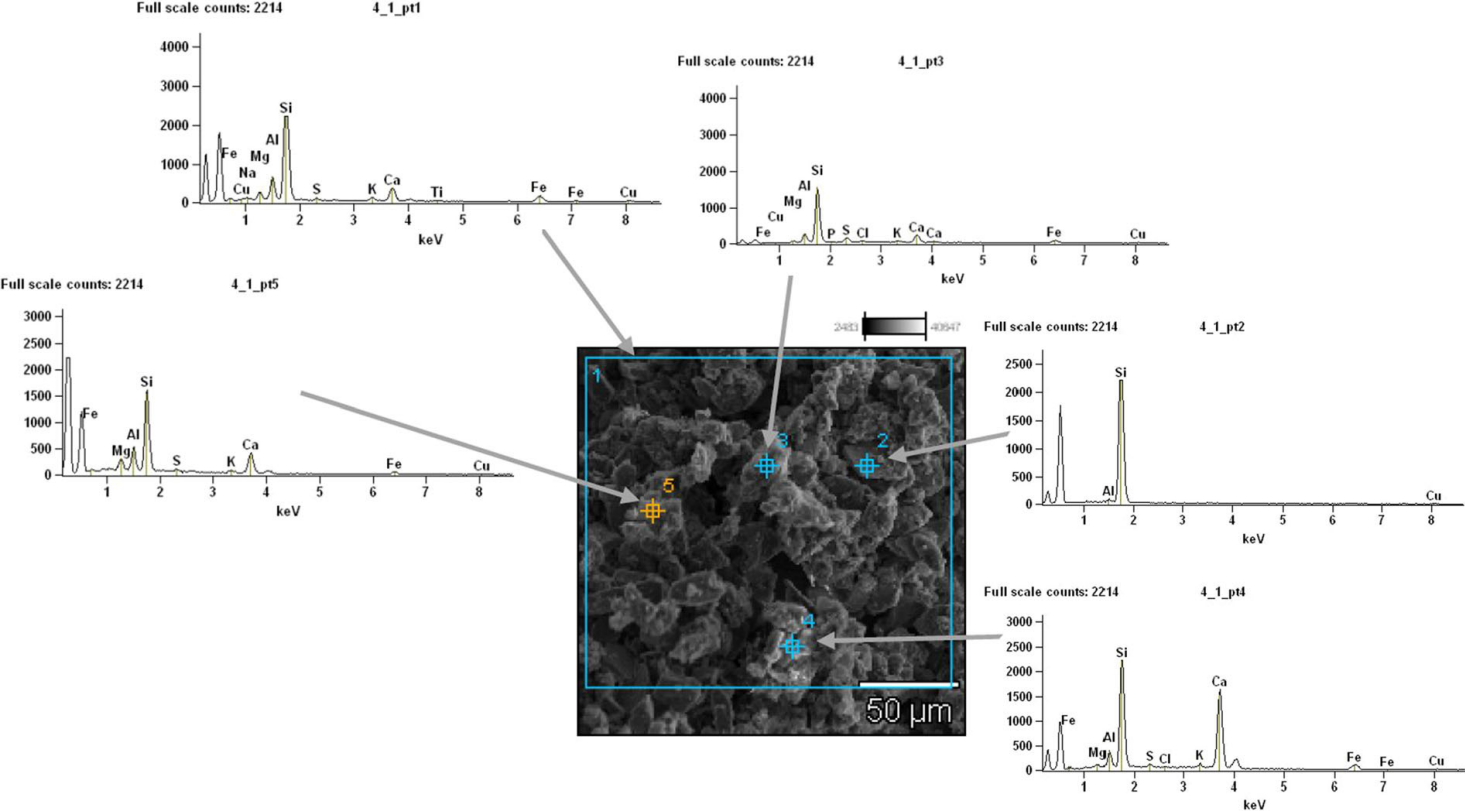
SEM-EDS image of motorway road dust (accelerating voltage = 15.0 kV; magnification = ×500)

**Fig. 5:**
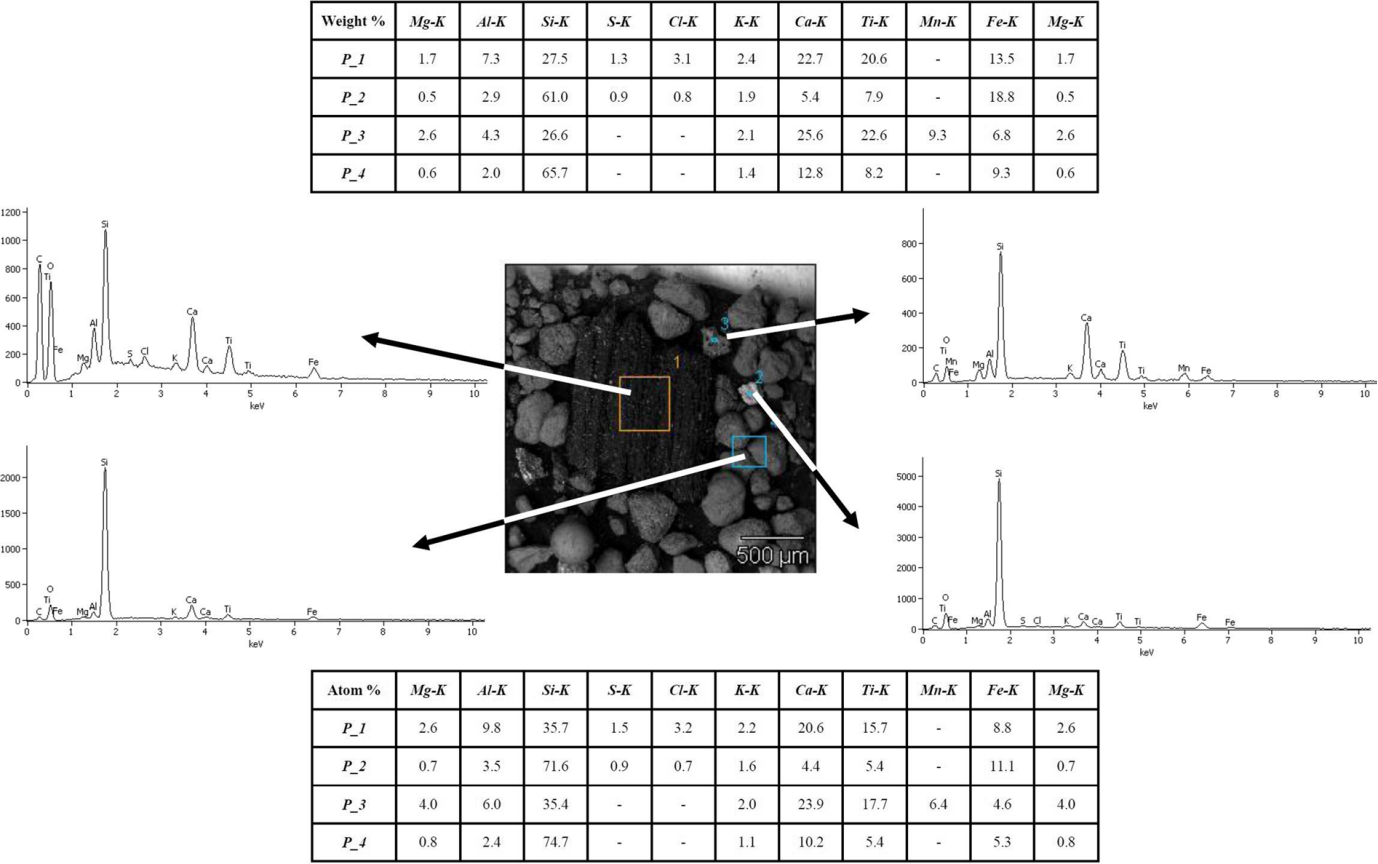
Results from SEM-EDS analyses of urban road dust (accelerating voltage = 15.0 kV; magnification = ×200)

The results from motorway and urban dusts were compared with mountain road dust, which was considered unpolluted control sample. Figures 4 and 5 present results from the qualitative chemical analysis of motorway and urban road dusts using SEM-EDS.

The concentrations of heavy metals in bulk samples of each of the examined road dust types are presented in Table 2. Urban and motorway road dust samples were highly contaminated with all of the investigated metals when compared with dust collected from the unpolluted mountain region. Urban road dust was significantly more contaminated with Zn, Pb, and Cu than motorway dust.

**Table 2:** Concentrations of heavy metals in bulk samples of motorway, urban, and mountain road dusts

Elements	Cr	Zn	Fe	Pb	Ni	Cu	Ti	Sr	Ba	Se	Cd	Pd
Mean concentration (mg/kg)												
Motorway dust (*n* = 3)	232	1420	42,038	321	62.3	198	2067	119	114	0.078	0.45	0.033
Urban road dust (*n* = 3)	211	2030	50,298	430	43.7	239	778	99.8	192	0.059	0.352	0.031
Mountain road (*n* = 1)	34.2	211	13,467	24.2	29.6	78.4	309	99.5	87.7	0.02	0.12	0.015

### Distribution of heavy metals within road dust size fractions

Mean concentrations of metals in the five analyzed fractions are presented in Table 3. The finest fraction (<20 μm) of motorway and urban dust samples was heavily contaminated with all of the investigated metals when compared with the other fractions. The <20-μm fraction of urban dust was almost twice as contaminated as motorway dust.

**Table 3:** Concentrations of heavy metals in the different size fractions of urban, motorway, and mountain road dusts

Mean concentration in fractions	Weight %	Cr	Zn	Fe	Pb	Ni	Cu	Ti	Sr	Ba	Se	Cd	Pd
Motorway dust mg/kg (*n* = 3)													
<20 μm	0.86	182	1829	51,907	456	109	287	2678	132	533	0.157	0.912	0.095
20–56 μm	1.58	132	1609	50,899	362	98.2	209	2289	129	302	0.109	0.659	0.087
56–90 μm	2.86	112	1390	46,758	326	87	120	1198	113	256	0.043	0.584	0.029
90–250 μm	25.72	109	593	30,145	213	49.9	93.2	399	108	99.8	0.056	0.245	0.023
>250 μm	68.98	111	730	19,940	138	48.2	78.2	422	109	107	0.053	0.098	0.019
Urban road dust mg/kg (*n* = 3)													
<20 μm^a^	0.94	219	2565	62,897	789	87.9	389	863	129	509	0.161	1.094	0.101
20–56 μm	2.98	198	2267	54,389	743	89.4	353	809	98.2	312	0.086	0.78	0.089
56–90 μm	6.87	169	1768	50,980	569	76.5	189	609	95.3	267	0.034	0.56	0.044
90–250 μm	32.98	222	1879	41,434	309	28.4	103	189	66.5	109	0.029	0.218	0.034
>250 μm	56.23	87.9	946	20,390	387	22.7	89	176	89.4	201	0.022	0.081	0.022
Mountain road dust mg/kg (*n* = 1)													
20–56 μm	0.54	51.7	559	14,098	188	54.8	209	498	87.3	161	0.032	0.192	0.016
56–90 μm	1.41	48.3	396	13,077	40.9	42.2	86.4	308	78.1	113	0.019	0.147	0.015
90–250 μm	28.31	23.9	221	12,900	47.6	28.1	35.3	297	79.4	79.9	0.028	0.111	0.013
>250 μm	69.74	38.9	198	10,890	12.4	23.3	33.9	288	134	89.9	b.d.l.	0.198	0.015

*b.d.l.* below detection limit

^a^ *n* = 1

Concentrations of metals in the 20–56- and 56–90-μm size fractions were also higher in urban dust than in motorway road dust. The greatest diversity in concentrations was found in the 90–250-μm fraction for motorway and road dusts. The results revealed very high concentrations of Zn in the motorway and urban roads. For example, in the <20-μm fraction, the mean Zn concentration was 2565 mg/kg on the urban road and 1829 mg/kg on the motorway.

Both types of road dust were especially contaminated with Zn, Cr, Cu, Pb, Fe, Ba, and Ti. On average, the dust collected from the urban area was 30 % more contaminated with Zn, Cu, Pb, and Fe than motorway dust. Motorway dust revealed Ti concentrations to be three times higher than those in urban dust. The concentrations of Cr, Ni, Sr, Ba, Se, and Cd in both road dust types were comparable and were significantly elevated when compared with the concentrations found in mountain road dust. Contamination with Zn can be attributed to the wear and tear of tires, because ZnO and ZnS are added to activate vulcanization in the tire tread. Cu contamination could originate from the frictional materials used in the brake system. Lead is an important component of bearing alloys, but significant concentrations of this metal in road dust could have also originated from the Silesia industrial region, which was located nearby. Until recently, lead was also used as a material for wheel balancing weights, but it has been replaced by zinc weights. It should be, however, noted that Pb is very persistent element and its elevated concentration in urban dust could also be a consequence of common use of PbO_4_ as gasoline additive in Poland up to March 2005. Contamination of road dust with chromium is the result of adding it as a main component to alloys used to produce wrist pins and connecting rods. An elevated amount of Ba in urban dust could be a consequence of using BaSO_4_ for improving wear resistance.

**Fig. 6:**
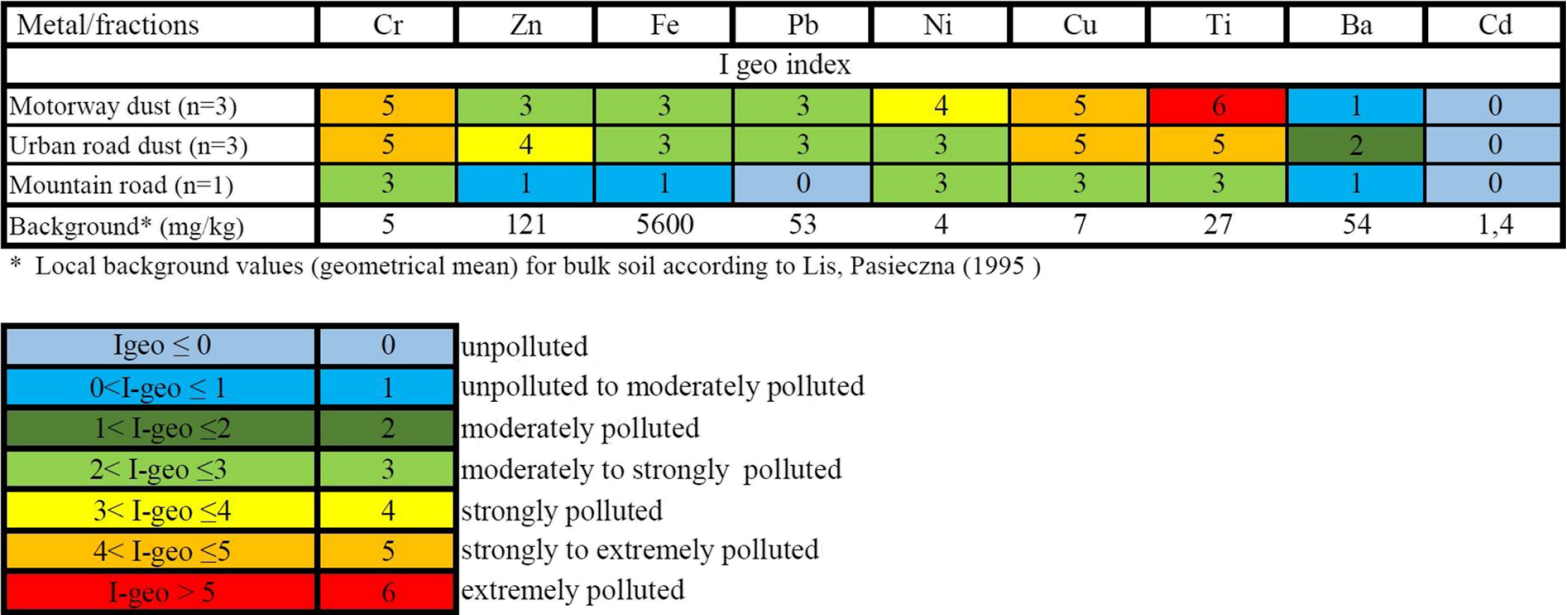
*I*_geo_ calculation for road dust (bulk samples)

**Fig. 7:**
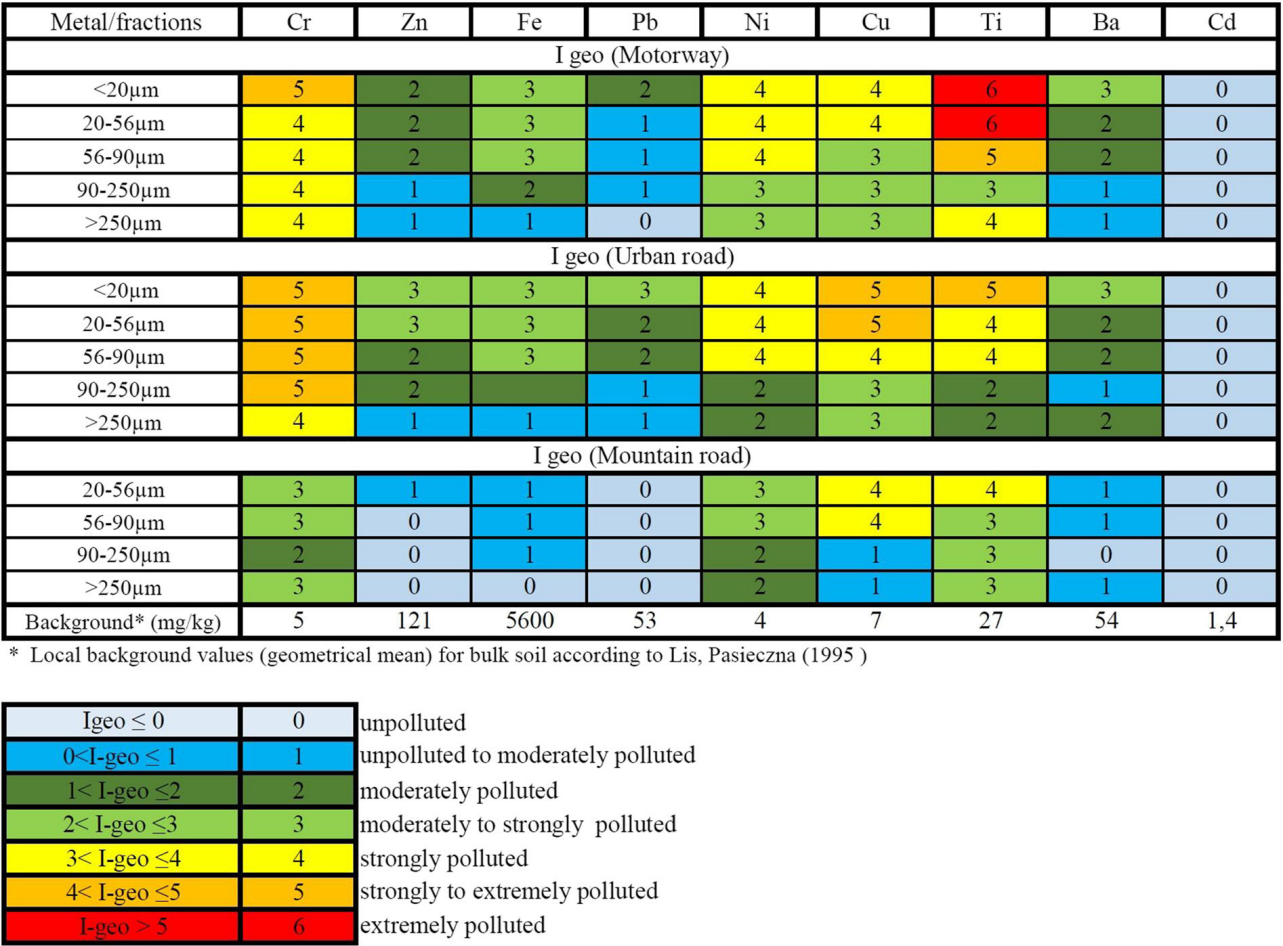
*I*_geo_ calculation for selected road dust size fractions

Concentrations of metals in the studied road dust were then used to calculate the *I*_geo_ accumulation index (Müller 1969), and the results are presented in Figs. 6 and 7.

Road dust contamination level was estimated based on *I*_geo_ index calculation. It was concluded that the finest fractions of motorway road dust (<20 and 20–56 μm) were extremely contaminated with Ti (class 6); strongly contaminated with Cr, Ni, and Cu (class 4); and moderately contaminated with Zn (class 2). As per the contamination with the remaining metals (Fe, Pb, Ba), the finest fractions of road dust were classified between 1 and 3 *I*_geo_ class (Fig. 7, Table 3). Furthermore, Ti concentration in motorway road dust (finest fractions) exceeds up to 1000 times the local background value for Upper Silesia region (according to Geochemical Atlas of Poland).

Calculation of *I*_geo_ index for urban road dust revealed that its finest fractions are strongly to extremely contaminated with Cu, Cr, and Ti (class 5) and strongly contaminated with Zn, Ni, and Pb (classes 3–4). Both road and urban dusts were not contaminated with Cd meeting class 0 according to *I*_geo_.

Detailed results of road dust analysis revealed that the finest fraction of both types of dust were the most contaminated ones (Fig. 7, Table 3). Contamination of the fraction less than 20 μm is likely a consequence of clay minerals present in the surrounding soil, but heavy metals in this fraction can also originate from dust derived from brake lining wear.

## Conclusions

Bulk samples and the finest fractions of urban and motorway dusts (up to 56 μm) were significantly contaminated with all of the investigated metals, in particular with Ti, Cu, Cr, Ni, Zn, Fe, Pb, and Ba. Since Ti, Cu, and Cr are well-recognized key tracers of non-exhaust brake wear emissions, high concentrations of these metals confirm that brake wear highly contributes to road dust contamination. Elevated concentration of Ti in both motorway and urban road dusts is undoubtedly of anthropogenic origin, and it might be linked to the use of alkali metal titanates as inorganic fillers for the purpose of stabilizing friction coefficient. Furthermore, contamination of road dust with copper is probably due to using it as a component of reinforcing fiber, which in form of chips or granules, combined with Zn, improves toughness and strength of brake pads.

The fine fraction of urban dust is more contaminated than the motorway dust. This is due to different driving conditions on urban roads and motorways. In the city, there is much more braking involved, what causes additional contamination of road dust with brake pads wear. Moreover, limited or poor air circulation in the city causes that resuspension of road dust occurs more likely. Sieve analysis of brake lining dust and tire dust did not reveal particles with diameters greater than 250 μm. From this, we have concluded that the fraction >250 μm in road dust was of geogenic origin and was not related to the dust from braking systems. High Zn concentrations in the coarse fraction (>250 μm) were likely the result of tire abrasion. This conclusion is in line with the findings of Gunawardana et al. (2012), who stated that even though individual ZnO particles are, on average, no bigger than 65 μm, in reality, they form agglomerates 1000–2000 μm in diameter. It should also be noted that the fine fraction can agglomerate with coarse fraction in other cases as well.

Particles <56 μm from brake linings and tire dust should be considered an indicator of heavy metal pollution because over 90 % of these metals are found in the fine fraction. Attention should also be drawn to the fact that significant sources of metals in the finest fractions of road dust could also be of geogenic origin.

On the mountain road, where traffic is significantly less intensive, vehicles had practically no influence on the level of dust contamination. Confirming this, the concentration of Pd, which comes from catalytic converters in vehicles, in mountain road dust was much lower in the finest fraction, when compared with motorway and urban dusts. Considering the geochemical background levels and *I*_geo_, it can be assumed that Cr, Zn, Pb, and Cu, which were detected in both types of road dust, pose a significant hazard to the environment.

Monitoring of the fine fraction of road dust should be intensified since this fraction easily enters the environment and human airways. Processes such as resuspension of road dust and exhaust and non-exhaust car emission mostly affect children or babies in strollers, because the highest concentrations occur low to the ground. It would also be valuable to determine speciation of different metals in urban and motorway dusts to better understand the health risks that they pose. Further studies on the impact of traffic-related emissions on human health should also be considered. The obtained results can furthermore be used for cost-benefit analyses.
